# Exendin-4–encapsulated dissolving microneedle arrays for efficient treatment of type 2 diabetes

**DOI:** 10.1038/s41598-018-19789-x

**Published:** 2018-01-18

**Authors:** Shayan Fakhraei Lahiji, Yoojung Jang, Inyoung Huh, Huisuk Yang, Mingyu Jang, Hyungil Jung

**Affiliations:** 1 Department of Biotechnology, Building 123, Yonsei University, 50 Yonsei-ro, Seoul, 03722 Seodaemun-gu Korea; 2 Juvic Inc., Building 102, Yonsei Engineering Research Park, 50 Yonsei-ro, Seoul, 03722 Seodaemun-gu Korea

## Abstract

Dissolving microneedles (DMNs) are microscopic needles capable of delivering encapsulated compounds and releasing them into the skin in a minimally invasive manner. Most studies indicate that encapsulating therapeutics in DMNs is an efficacious approach; however, the importance of evaluating the activity of encapsulated compounds, during the fabrication process, has not been examined in detail. Conducting an analysis of thermal, chemical, and physical stress factors, including temperature, pH, and the interaction of the polymer and therapeutics mixture during preparation, is essential for retaining the activity of encapsulated therapeutics during and after fabrication. Here, we optimised the thermal, chemical, and physical parameters for the fabrication of exendin-4 (Ex-4)–encapsulated DMNs (Ex-4 DMNs). Ex-4, a peptide agonist of glucagon-like peptide (GLP) receptor, is used for glycaemic control in patients with type 2 diabetes. Our findings indicate that optimising the parameters involved in DMN fabrication retained the activity of Ex-4 by up to 98.3 ± 1.5%. Ex-4 DMNs reduced the blood-glucose level in diabetic mice with efficiency similar to that of a subcutaneous injection. We believe that this study paves way for the commercialisation of an efficient and minimally invasive treatment for patients with type 2 diabetes.

## Introduction

Type 2 diabetes, also known as non-insulin-dependent diabetes mellitus, disrupts the normal action and secretion of insulin in the body^1^. This can lead to a series of severe syndromes such as cardiovascular disease, hypertension, stroke, blindness, and increased risk of cancer^2^. Exendin-4 (Ex-4), a 39-amino acid incretin hormone that acts as a glucagon-like peptide-1 receptor agonist (GLP-1 agonist), is used for glycaemic control in patients with type 2 diabetes^2,3^. Long-term treatment with Ex-4 reduces food intake, lowers gastric motility^4^, and suppresses glucagon secretion^5^, without causing severe side effects, in patients with type 2 diabetes^6^.

Ex-4 is generally administered by subcutaneous (SC) injection on a daily basis, which may result in pain, needle phobia, infection, and inconvenience to the patient^7^. Daily injections create a large amount of needle waste, which may result in needle-stick injury, blood-borne virus transmission, and needle recycling costs^8–10^. To improve patient compliance and delivery efficacy of Ex-4, various studies have evaluated the encapsulation of Ex-4 in microspheres, long-acting conjugate polymers, and nanoparticles^11–14^. However, due to concerns about stability and toxicity, hypodermic injection remains the most common delivery route for patients with type 2 diabetes. Therefore, the development of a safe, painless, and efficient delivery system for Ex-4 is important for improving patient compliance and overcoming limitations associated with SC injection^15^.

Dissolving microneedles (DMNs), which consist of a biodegradable polymer and therapeutic, are used for delivering encapsulated compounds, after insertion into the skin, in a minimally invasive manner^16,17^. DMNs provide an efficient and accurate delivery system for encapsulated therapeutics, and overcome the limitations of hypodermic injections^18^. Most studies suggest that DMNs can be used as a rapid and efficient transdermal delivery system for small-molecule drugs, nanoparticles, and biomolecules such as insulin, influenza vaccine, and the human growth hormone^19–23^. Despite this potential for using DMNs as highly efficient delivery vehicles for Ex-4, only a few reports have evaluated the encapsulation of Ex-4 in DMNs (Ex-4 DMNs)^24,25^. Furthermore, these studies have mainly focussed on the design and delivery of Ex-4 DMNs rather than addressing the activity of encapsulated Ex-4 during the fabrication process and storage period. Therefore, we hypothesised that optimising the parameters involved in DMN fabrication will help maintain the activity of Ex-4 at a high level. Optimising such a minimally invasive system will improve compliance of patients with type 2 diabetes and help commercialise Ex-4 DMNs as an efficient replacement for the currently used SC.

In this study, we used the most recent technique for DMN fabrication, in which the activity of encapsulated compounds is highly conserved during the fabrication process^26^. We analysed the thermal, chemical, and physical factors that can affect the activity of Ex-4 DMNs. Based on these data, we optimised the fabrication conditions for Ex-4 DMNs for fabrication of Ex-4 DMNs in which the activity of encapsulated Ex-4 can be maintained at up to 98.3 ± 1.5%. The *in vivo* delivery comparison of SC injection and Ex-4 DMNs suggested that DMNs possess efficiency similar to that of SC in reducing the levels of blood glucose. We believe that compared with the current delivery systems, Ex-4-DMNs provide efficient delivery and can improve patient compliance, thereby offering an effective alternative for treating patients with type 2 diabetes.

## Results and Discussion

### Effect of temperature and pH on the activity of Ex-4

**Figure 1:**
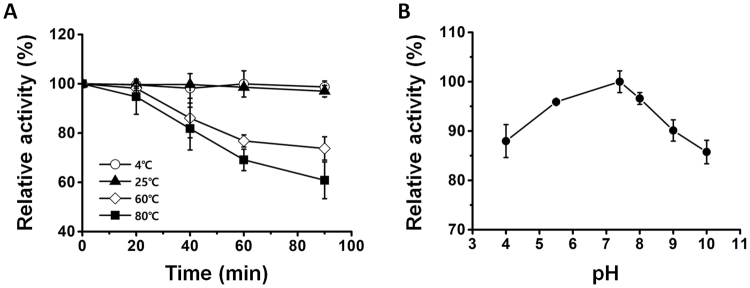
Effects of temperature and pH on the activity of Ex-4. (**A**) The activity of Ex-4 was assessed at 4, 25, 60, and 80 °C for up to 90 min. At 90 min post-evaluation, the activity of Ex-4 was not affected at 0 and 25 °C, whereas it was greatly reduced at 60 and 80 °C. (**B**) Changes in the pH affect the activity of Ex-4; the activity of Ex-4 was most retained at neutral pH.

Temperature and pH can affect the activity of encapsulated compounds during the process of DMN fabrication^27^. Therefore, we evaluated the effects of thermal conditions on the activity of Ex-4 at 4, 25, 60, and 80 °C for up to 90 min (Fig. 1A). The results indicate that the activity of Ex-4 was maintained for up to 90 min at 4 and 25 °C; the activity was significantly reduced at 60 and 80 °C to 73.7 ± 4.7% and 60.8 ± 7.4%, respectively. These results show that, although the activity of Ex-4 was maintained at a moderate temperature during fabrication, increasing the temperature greatly reduced the activity of Ex-4. Therefore, to prevent the possible loss in the activity of Ex-4 during DMN fabrication, it is necessary to maintain the thermal conditions below 25 °C.

The pH of the polymer, which is the therapeutic mixture used in the fabrication of DMNs, may also affect the activity of encapsulated therapeutics^28^. We assessed the activity of Ex-4 at the pH range of 4 to 10 to find the optimal pH for retaining the activity of Ex-4. Our results indicate that Ex-4 retained its activity at neutral pH, whereas its activity decreased to 87.9 ± 3.3% at pH 4 and 85.7 ± 2.3% at pH 10 (Fig. 1B). These findings are in agreement with those of previous studies, showing that most proteins are denatured under acidic and basic conditions^29^. Based on these results, we conclude that maintaining a neutral pH is crucial to avoiding loss in the activity of Ex-4 during preparation of the solution, which is performed before to the fabrication of DMNs.

### Effects of polymer concentration on the pH levels and activity of Ex-4

**Figure 2:**
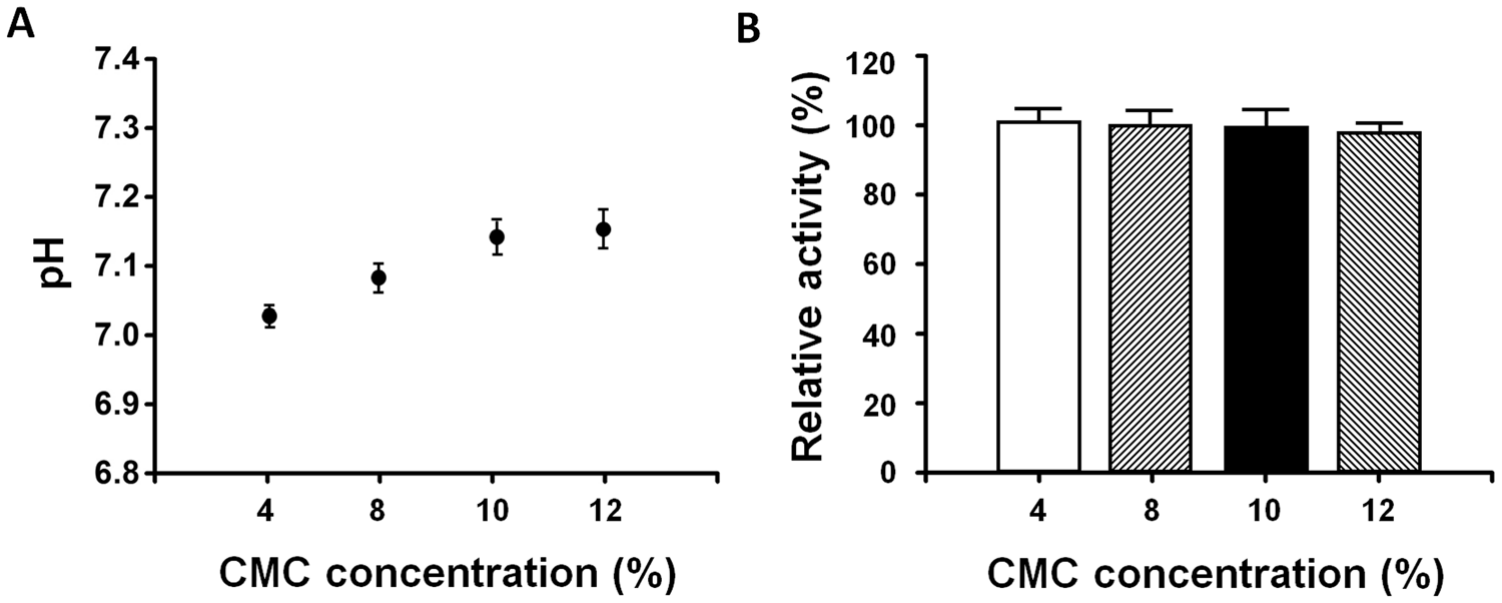
The effects of CMC concentration on the pH and activity of Ex-4. (**A**) The pH of CMC was gradually increased by increasing the concentration of CMC from 4 (7.02 ± 0.02) to 12% (7.12 ± 0.06) (w/v) (n = 3). (**B**) The activity of Ex-4 in the mixture was not affected at the concentrations of 4, 8, 10, and 12% CMC (w/v) (n = 3).

A DMN array is fabricated by mixing a biodegradable matrix with therapeutics and solidifying this mixture into sharp micro-structures. Addition of a biodegradable polymer such as Ex-4 to therapeutics, however, may change the pH of the mixture, leading to aggregation^30,31^ and irreversible inactivation of Ex-4^32^. To fabricate DMNs, in this study, we selected CMC, a widely-used, FDA-approved backbone matrix material; we then evaluated the changes in pH at CMC concentrations of 4, 8, 10 and 12% (w/v) (Fig. 2A). Our findings show that the pH of CMC, at the concentrations of 4 to 12%, ranged from 7.02 ± 0.02 to 7.12 ± 0.06, respectively. The pH range of CMC, at different concentrations, was at a neutral level; therefore, the activity of Ex-4, in the polymer mixture, was not affected, which is in agreement with the results shown in Fig. 1B.

To confirm this, and to find the optimal concentration of CMC at which the activity of Ex-4 is retained the most, we examined the effects of CMC concentration on the activity of Ex-4. As expected, our results indicate that the activity of Ex-4 was retained at all the tested concentrations of 4, 8, 10 and 12% CMC (w/v) (Fig. 2B). Overall, these findings show that as long as pH was maintained at a neutral level, the activity of Ex-4 was retained regardless of the CMC concentration. However, it is important to note that the results, obtained in this experiment, cannot be generalised to other polymers. Each polymer may react differently with Ex-4 in the mixture and, therefore, should be evaluated separately to ensure that it does not affect the activity of Ex-4.

### Fabrication of Ex-4 DMNs

**Figure 3:**
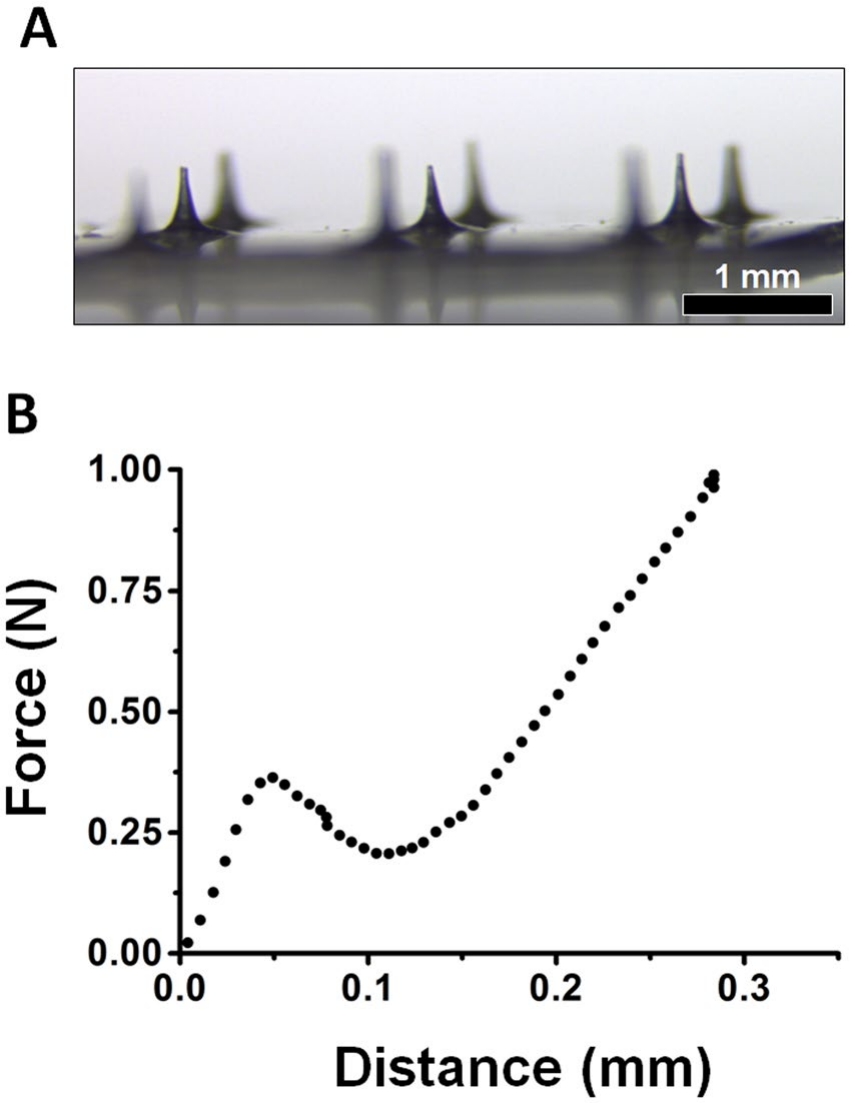
Fabrication of Ex-4 DMNs and fracture force analysis. (**A**) Image of a 3 × 3 array of Ex-4 DMNs having the length of 450 ± 25 μm and tip diameter of 35 ± 5 μm. (**B**) Fracture force analysis of Ex-4 DMNs indicates that the DMNs possess sufficient mechanical strength (0.36 ± 0.09 N) to penetrate the skin without breakage (n = 3).

To fabricate Ex-4 DMNs, capable of penetrating the skin without breakage, it is crucial to maintain a mixture viscosity of 0.75 to 3 Pa.s^33,34^. We found that CMC concentrations of 4 and 8% were not suitable for the fabrication of DMNs, whereas both 10 (1.47 ± 0.15 Pa.s) and 12% (2.75 ± 0.09 Pa.s) possessed the required viscosity (Supplementary Fig. 1). Conversely, increasing the concentration of the polymer slows the diffusion of encapsulated Ex-4 into the skin^21^. Therefore, we selected 10% as the optimal concentration of CMC for DMN fabrication. The Ex-4 DMNs were fabricated with a length of 450 ± 25 μm and tip diameter of 35 ± 5 μm, in 3 × 3 arrays, as shown in Fig. 3A (10 μg Ex-4/DMN patch). To confirm that the fabricated DMNs can successfully penetrate the skin barrier without breakage, we analysed the fracture force of Ex-4 DMNs. The fracture force of a single Ex-4 DMN was 0.36 ± 0.09 N, which is higher than the minimum force required for skin penetration per DMN (Fig. 3B)^35^. Altogether, these data indicate that among the evaluated concentrations, 10% CMC was the optimal concentration for fabricating DMNs, possessing sufficient viscosity to provide DMNs with mechanical strength required to penetrate the skin.

### *Ex vivo* dissolving and release profile of Ex-4 DMNs

**Figure 4:**
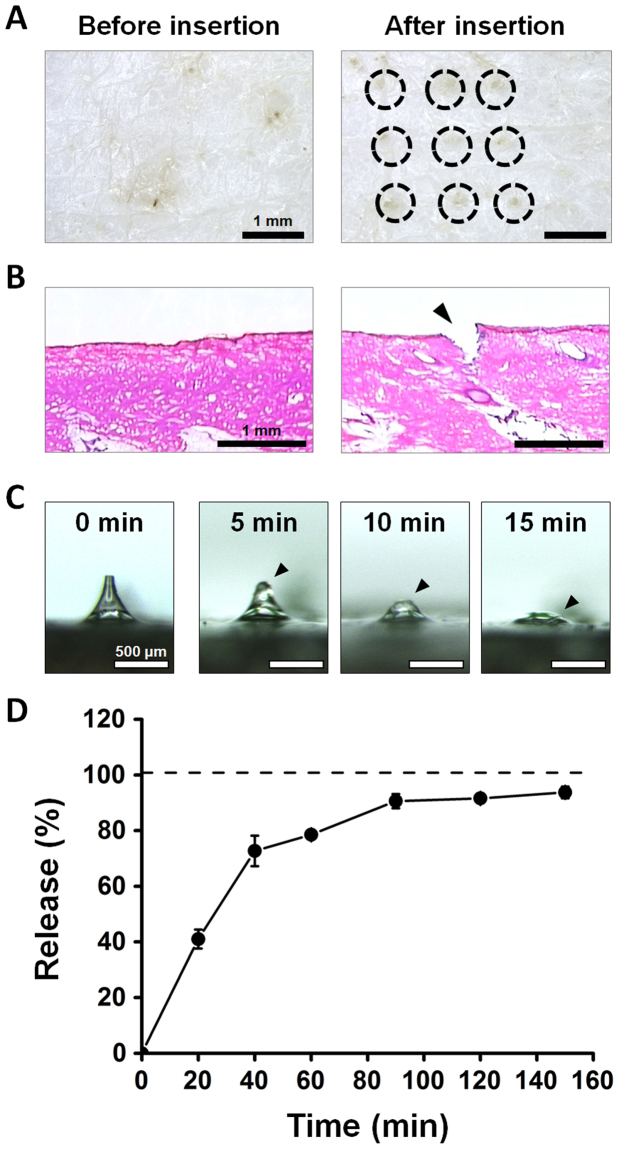
Inserting Ex-4 DMNs into pig cadaver skin. (**A**) The 3 × 3 channels were created in pig cadaver skin after applying the Ex-4 DMN patch. Insertion spots are indicated by a dashed line. (**B**) Histological analysis of pig cadaver skin upon application of a single Ex-4 DMN. Arrow indicates insertion spot. (**C**) Morphological changes in Ex-4 DMNs at 0, 5, 10, and 15 min post-application. Ex-4 DMNs were almost completely dissolved 15 min post-application. Arrows indicate DMNs. (**D**) The release profile of Ex-4 DMNs, during their transit through pig cadaver skin, was measured via a Franz diffusion cell for up to 150 min post-application. Data show that 93.7 ± 2.1% of Ex-4 was delivered to the chamber at 150 min post-application.

To assess the skin insertion capability of Ex-4 DMNs *ex vivo*, the arrays were inserted into pig cadaver skin, and the insertion spots were imaged post-application (Fig. 4A). The 3 × 3 channels, created on the cadaver skin, confirmed the results of the fracture force analysis, indicating that Ex-4 DMNs penetrated the skin without breakage. In addition, we performed histological analysis of pig cadaver skin upon application of a single Ex-4 DMN. Results showed that Ex-4 DMNs could successfully pierce and penetrate into the pig cadaver skin (Fig. 4B). To confirm that the DMNs had completely dissolved upon application, we used bright-field light microscopy to examine the morphological changes in Ex-4 DMNs at 5, 10, and 15 min post-application. Our data indicate that the DMNs were almost completely dissolved at 15 min post-application into pig cadaver skin (Fig. 4C).

We used the Franz diffusion cell, which mimics the natural blood circulation in the body, to assess the release profile of Ex-4 DMNs through the skin^36^. Although our data, in Fig. 4B, show that Ex-4 DMNs were completely dissolved 15 min post-application, only 41 ± 3.4% of the encapsulated Ex-4 content was detected in the chamber of the diffusion cell at 15 min (Fig. 4D). We assume this time gap is caused by the barrier properties of the skin, which slow the permeation of Ex-4 through the skin, thereby affecting its transit to the chamber. At 150 min post-application, 93.7 ± 2.1% of Ex-4 reached the chamber. The amount of Ex-4, which remained undelivered 150 min post-application, may have resulted from incomplete insertion of the DMNs, which is unavoidable in patch-based DMNs^37^. Previous studies suggest that by utilising an applicator, it is possible to overcome the incomplete insertion of DMNs into the skin^37^. Blank DMNs (without Ex-4) were used as controls and were not detected for up to 15 min post-insertion (data not shown). These findings show that the DMNs, fabricated in this study, can efficiently deliver encapsulated Ex-4 through the skin barrier.

### Activity of Ex-4 post DMN fabrication

**Figure 5:**
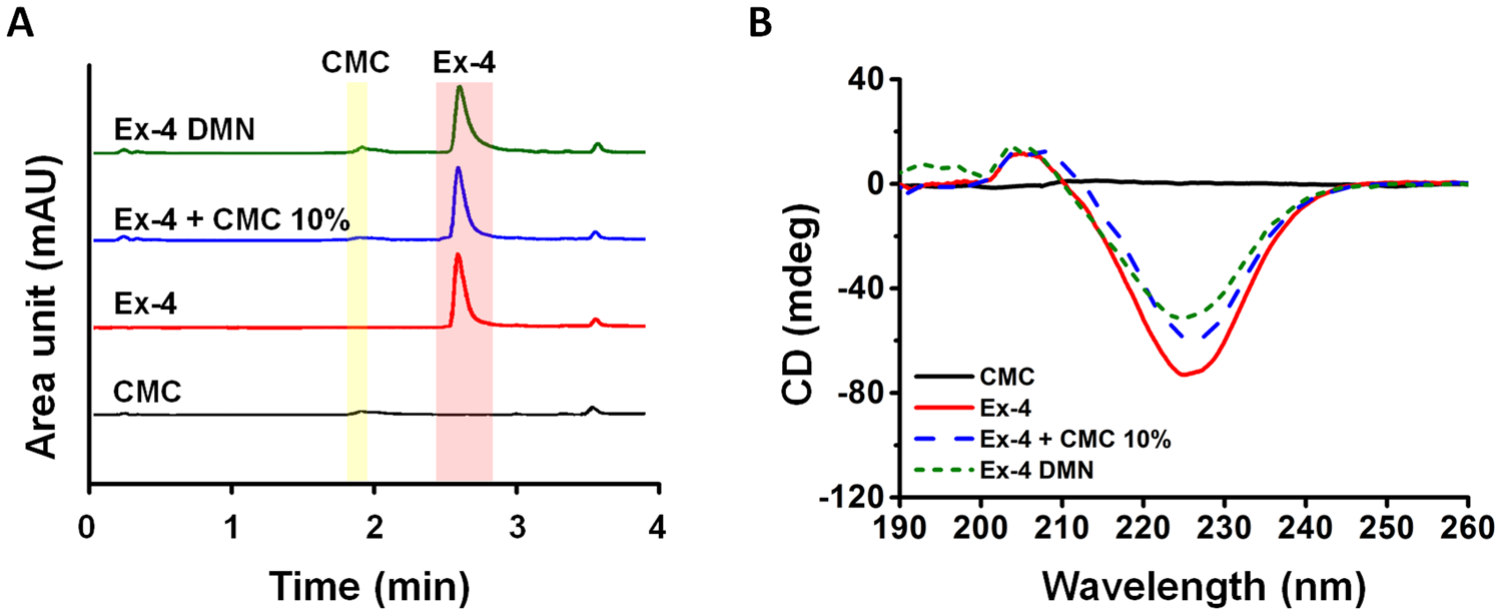
Evaluation of the activity of Ex-4 DMNs via UPLC and CD. (**A**) UPLC analysis indicated that Ex-4 retains its activity post DMN fabrication. (**B**) CD spectra of CMC, Ex-4, Ex-4–CMC at 10%, and extract of Ex-4 DMN. CD analysis showed the same retention time for Ex-4 in both Ex-4–CMC at 10% and Ex-4 DMN without any changes in the secondary structure of Ex-4 after DMN fabrication.

To evaluate the activity of Ex-4 DMNs post-fabrication, we used ultra-performance liquid chromatography (UPLC) to compare the initial activity of Ex-4 in 10% CMC (before DMN fabrication) with that of extracts of Ex-4 DMN (after DMN fabrication) (Fig. 5A). The retention times of 10% CMC and Ex-4 peaked at 1.85 and 2.49 min, respectively, without any significant shifts in all groups. These results confirm that the activity of Ex-4 was well maintained during the fabrication process.

The unique secondary structure of the peptides is responsible for their biological activity and functionality^38^. Therefore, we examined the secondary structure of Ex-4, before and after DMN fabrication, using circular dichroism (CD). The Ex-4 DMN group showed the same peak as that of Ex-4, with a negative spectra range of 210–247 nm. The area under the curve of 10% mixture of Ex-4 CMC, and that of Ex-4 DMN extract, were smaller than that of Ex-4 (Fig. 5B). Although additional experiments are required to confirm our assumption, this may be because CMC prevents CD from accurately detecting Ex-4. CD analysis confirmed that there were no significant changes in the secondary structure of Ex-4 post DMN fabrication, which was consistent with the results obtained by UPLC.

### Cytotoxicity and stability of Ex-4 DMNs during storage

**Figure 6:**
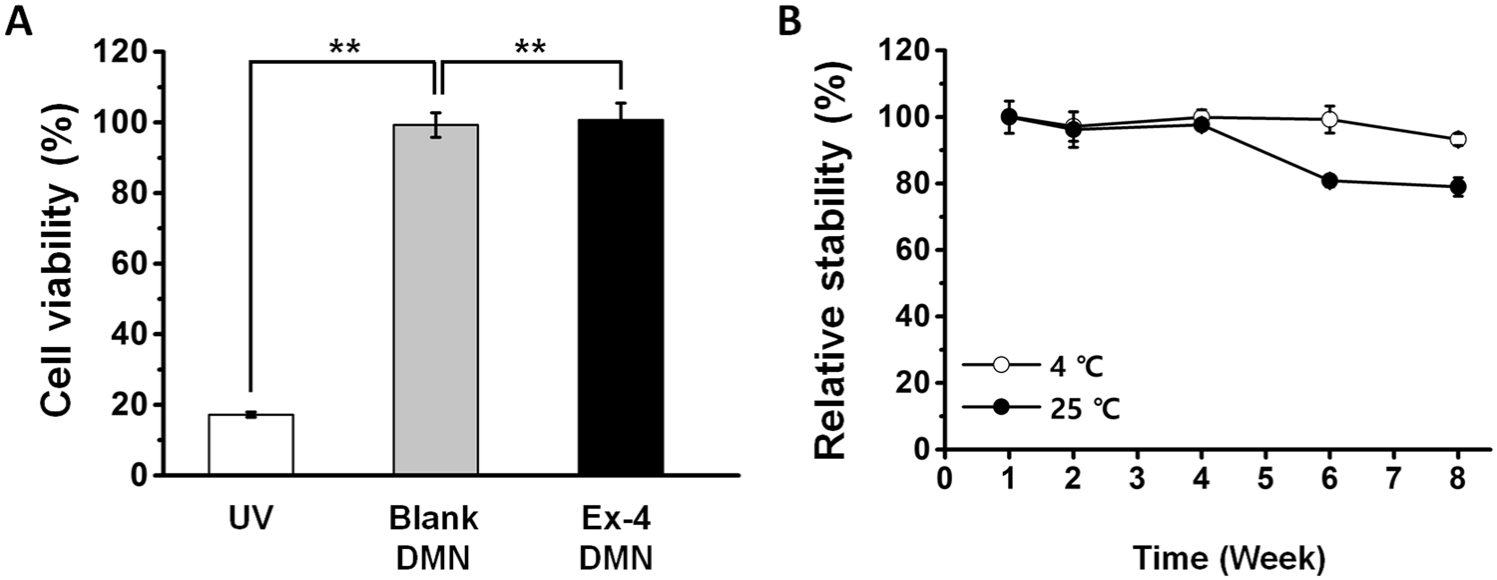
Cytotoxicity and storage stability of Ex-4 DMNs. (**A**) Treatment with blank DMNs and Ex-4 DMNs does not induce cytotoxicity in HEK293T cells (n = 3, P < 0.01). (**B**) The activity of Ex-4 DMNs, at 8 weeks post-storage at 4 °C, is gradually reduced to 93.2% ± 1.8; at 8 weeks post-storage at 25 °C, the activity of encapsulated Ex-4 is greatly reduced to 78.9% ± 2.8 (n = 3).

To assess the safety of Ex-4 DMNs for *in vivo* application, we used HEK293T cells to conduct a cytotoxicity analysis of the patches (Fig. 6A). HEK293T cells, treated with ultraviolet (UV) radiation, at 17.2 ± 0.7% viability, were used as negative controls for comparison with Ex-4 DMN-treated cells. Treatments using extracts of blank DMNs (CMC only) and Ex-4 DMNs showed viabilities of 99.3 ± 3.5% and 99.7 ± 5.8%, respectively. These results suggest that Ex-4 DMN patches are safe for *in vivo* application and do not show significant cytotoxicity.

To optimise the conditions for storing Ex-4 DMNs, we evaluated the activity of Ex-4, for up to 8 weeks, at the temperatures of 4 and 25 °C (Fig. 6B). At 4 weeks post-storage, the activity of Ex-4 was well retained at 99.9 ± 2.2% and 97.6 ± 1.27% at 4 and 25 °C, respectively. At 8 weeks, however, the activity of Ex-4 was reduced to 93.2 ± 1.8% and 78.9 ± 2.8% at 4 and 25 °C, respectively. These findings indicate that storing Ex-4 DMNs at low temperature is crucial for maintaining its biological activity and functionality. In addition to thermal conditions, we suggest that optimising packing methods, such as including a desiccant, or removing air before sealing, may increase the storage stability of encapsulated Ex-4.

### *In vivo* bioactivity of Ex-4 DMNs

**Figure 7:**
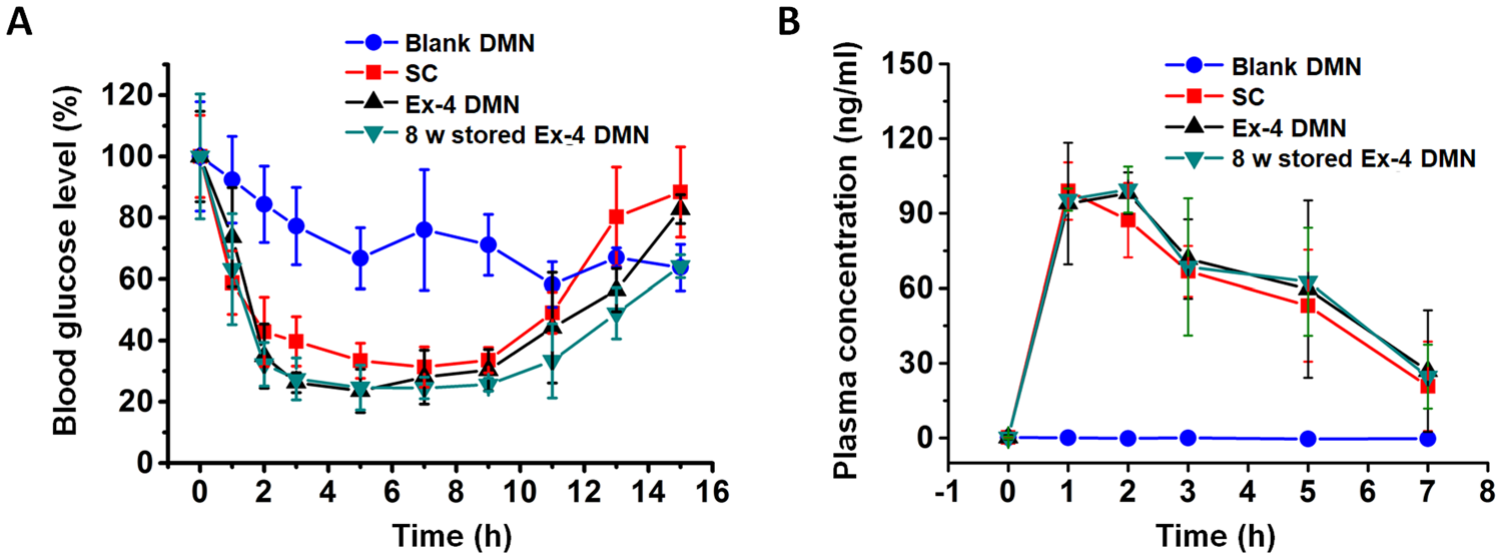
*In vivo* delivery efficiency of Ex-4 DMNs. (**A**) Blood-glucose levels in mice treated with blank DMNs, SC, Ex-4 DMNs, and Ex-4 DMNs stored for 8 weeks. Data indicate that Ex-4 DMNs can reduce the blood-glucose level with an efficiency similar to that of SC injection (n = 3). (**B**) The pharmacokinetics of Ex-4 in diabetic mice show that efficiency of delivery is similar for SC, Ex-4 DMNs, and Ex-4 DMNs stored for 8 weeks (n = 3).

Next, we examined the ability of Ex-4 DMNs to reduce the levels of blood glucose. For this, we compared Ex-4 DMNs with SC injection by delivering 10 μg of Ex-4 per group to groups of diabetic mice. At 3 h post-treatment, blood-glucose levels were reduced to 39.68 ± 8.05%, 26.26 ± 3.23%, and 27.46 ± 6.83% in the group treated with SC injection, that treated with Ex-4 DMN, and that treated with Ex-4 DMN stored for 8 weeks, respectively. The level of blood glucose in mice from the treated groups returned to basal levels after nearly 16 h (Fig. 7A). As expected, glucose levels were not decreased by treatment with blank DMNs. Interestingly, both Ex-4 DMN, and Ex-4 DMNs stored for 8 weeks, reduced the blood-glucose levels of diabetic mice with efficiency similar to that of SC injection. Altogether, these data suggest that DMNs can be as effective as SC injection in reducing blood-glucose levels, and that in the near future, Ex-4 DMNs can be used to potentially replace SC injection for patients with type 2 diabetes.

The plasma concentration of Ex-4, in treated diabetic mice, was measured for up to 7 h post-treatment (Fig. 7B). The maximum plasma concentration (Cmax) was 98.9 ± 11.5 ng/ml, 98.1 ± 8.3 ng/ml, and 99.6 ± 9.2 ng/ml in the groups treated with SC, Ex-4 DMNs, and Ex-4 DMNs stored for 8 weeks, respectively. The time, at which Cmax was attained (Tmax), peaked after 1 h in the SC group, whereas the groups treated with Ex-4 DMNs and Ex-4 DMNs stored for 8 weeks showed the peaks at 2 h post-application (Fig. 7B). This was likely caused by slower diffusion of DMNs compared with that of SC injection. As expected, no changes were observed in the plasma of mice treated with blank DMNs. Overall, the analysis of plasma concentration of Ex-4 confirmed that Ex-4 DMNs can effectively deliver encapsulated Ex-4 with effectiveness highly similar to that of SC injection.

### Conclusion

In conclusion, we have successfully developed and fabricated Ex-4 DMNs by optimising the thermal, chemical, and physical factors involved in DMN fabrication; these factors (temperature during fabrication, pH, and concentration of the polymer) were highly instrumental in maintaining the activity of encapsulated Ex-4. We selected 10% CMC because its viscosity afforded the fracture force of 0.36 N and because it rapidly dissolved inside the skin 15 min post-application. With respect to storage, Ex-4 DMNs showed no significant reduction in activity after 8 weeks of storage at a low temperature. Our findings indicate that Ex-4 DMNs can potentially replace the currently used SC injections, because they can efficiently reduce blood-glucose levels in patients with type 2 diabetes and are convenient to use. In addition to introducing a novel method for delivering Ex-4, our results highlight the importance of evaluating factors that may affect the activity of encapsulated therapeutics during each step of the DMN fabrication process.

## Methods

### Materials

Ex-4 (C_184_H_282_N_50_O_60_S) was purchased from the AnyGen Co. (Gwangju, Korea). Biodegradable polymer carboxymethylcellulose (CMC; 90 kDa), phosphate buffered saline (PBS, pH 7.4), HEPES buffer (pH 5.5), buffer solution (pH 4, 8, 9 and 10), potassium bromide (KBr), formic acid solution, acetonitrile (ACN), 4, 5- dimethylthiazol-2-yl-2,5- diphenyl tetrazolium bromide (MTT), and Avertin (2,2,2-tribromoethanol) were purchased from Sigma-Aldrich (St. Louis, MO, USA). Human embryonic kidney 293 T (HEK293T) cells were purchased from ATCC (Manassas, VA, USA). Male C57BLKS/J db/db mice (6 to 7 weeks old, 20 to 25 g) were purchased from Japan SLC, Inc. (Hamamatsu, Japan). The Ex-4 enzyme-linked immunoassay (ELISA) kit was purchased from Phoenix Pharmaceuticals, Inc. (EK-070-94; Burlingame, CA, USA).

### UPLC

UPLC, used to analyse the relative activity of Ex-4, was performed using an Agilent 1290 Infinity LC system (Agilent technologies, Santa Clara, CA, USA) equipped with an Eclipse Plus C18 RRHD column (2.1 mm × 50 mm, 1.8 μm particle size). Chromatographic separation was achieved using the mobile phases (A) 0.1% formic acid in deionised water and (B) 0.1% formic acid in acetonitrile, at the following gradient elution: 0.0–1.0 min, A 95%, B 5%; 1.0–3.0 min, A 95-35%, B 5–65%; 3.0–3.5 min, A 35%, B 65%; 3.5–3.8 min, A 35–95%, B 65-5%. The conditions for the analysis were set at a flow rate of 0.45 ml/min, injection volume of 10 μl, and column temperature at 25 °C with the detection wavelength of 276 nm.

### Effect of temperature and pH on the activity of Ex-4

Ex-4 was dissolved in PBS (pH 7.4) at the concentration of 100 μg/ml and incubated in a 4, 25, 60, and 80 °C water bath for 0–90 min to assess the effects of temperature. To evaluate the effects of pH on the activity of Ex-4, the Ex-4 solution, at the concentration of 100 μg/ml, was stored for 6 h at 4 °C in buffers at pH 4, 5.5, 7.4, 8, 9, and 10. The relative activity of Ex-4 was measured, via UPLC, with a standard curve obtained using the concentration of 0–1 mg/ml Ex-4 with R squared = 0.99. Ex-4 dissolved in PBS (pH 7.4) buffer was set as 100% active.

### pH and viscosity of the CMC mixture

To prepare the CMC mixture, powdered CMC was dissolved in PBS at the concentrations of 4, 8, 10, and 12% (w/v). These solubilised mixtures were then homogenised, via centrifugation, for 10 min at 4 °C and 4500 rpm using an Allegra X-30R centrifuge (Beckman Coulter, Palo Alto, CA, USA). The pH of each CMC mixture was measured using an ST3100 pH meter (Ohaus, Parsippany, NJ, USA). The viscosity of each CMC mixture was measured via a Viscometer (Rheosys, Princeton, NJ, USA) at the shear rate of 200/s, at 25 °C.

### Interaction of Ex-4 and the CMC mixture

To analyse the interaction of CMC and Ex-4, 10 mg/ml of Ex-4 was mixed with the 4, 8, 10, and 12% (w/v) CMC polymer, and examined by a Vertex70 FT-IR spectroscope (Bruker, Billerica, MA, USA). All the samples were measured using potassium bromide (KBr) pellets. Transmittance spectra were acquired in the range of 400–4000 cm^−1^ by accumulating 32 scans at a resolution of 4 cm^−1^. The bioactivity of Ex-4 was measured using an ELISA assay kit according to the manufacturer’s instructions. The mixture solutions of CMC and Ex-4 were diluted at the concentrations of 0.01 to 100 ng/ml, and the absorbance was calculated using a Victor X5 microplate reader (Perkin Elmer, Waltham, MA, USA) at 560 nm.

### Ex-4 DMN fabrication and fracture force analysis

Ex-4 DMN was fabricated in 3 × 3 arrays using 10 mg/ml of Ex-4 mixed with 10% CMC (w/v) via centrifugal lithography^26^. Blank DMNs, without Ex-4, were also fabricated using the same method and imaged via LEICA M165 FC (LEICA, Wetzlar, Germany). The mechanical fracture force of a single DMN was measured by a force test machine (Z0.5TN, Zwick/Roell, Ulm, Germany). DMN was attached to the stainless-steel station, and the sensor probe was pressed against the DMN at a speed of 3.6 mm/min at 0.02 N via an axial force.

### *In vitro* insertion analysis

A single Ex-4 DMN was applied to hairless pig cadaver skin (1 mm thick 2.5 × 2.5 cm^2^ sections) and stored at −10 °C until analysis. Tissue samples were first embedded in OCT compound (Tissue-Tek, Sakura Finetek, CA, USA) at −30 °C, and then sectioned at 25 μm per slice. The sections were next stained with hematoxylin for 5 min, eosin for 1 min, dehydrated through a series of alcohol gradients and cleared by xylene (Fisher Scientific, NH, USA).

### *In vitro* profile of Ex-4 release from DMNs

The Ex-4 DMN patches were applied to hairless pig cadaver skin (1 mm thick 2.5 × 2.5 cm^2^ sections) for 5, 10, and 15 min. Images of DMNs were acquired using a LEICA M165 FC microscope. The profile of Ex-4 release from DMNs was examined using a Franz diffusion cell (Hanson, Chatsworth, CA, USA). The diffusion cell was filled with 7 ml PBS (pH 7.4); then, the Ex-4 DMN patches were inserted into the hairless pig cadaver skin. After securing with a pinch clamp, the contents of the chamber were thoroughly mixed via a magnetic stirrer at 500 rpm, and 2 μl samples were acquired from the receptor at 0, 20, 40, 60, 90, 120, and 150 min. The amount of Ex-4 was quantitated by a NanoVue UV/Visible spectrophotometer (GE Healthcare, Little Chalfont, UK) at the absorbance of 208 nm. The value of the blank DMN was used as negative control.

### *In vitro* activity of Ex-4

The activity of Ex-4 (at the concentration of 100 μg/ml), that of a mixture solution of Ex-4 and 10% CMC (w/v), and that of the extract of Ex-4 DMN was examined via UPLC and CD at different stages of the DMN fabrication process. CMC (10% w/v) was used as negative control. CD was conducted using a Jasco CD Spectrophotometer J-815 (Jasco, Tokyo, Japan) with a path length of 10 mm. The parameters were set at a scanning speed of 100 nm/min, band-width of 0.1 nm, wavelength range of 190–260 nm, and the baseline was corrected using a blank solvent.

### Cytotoxicity of Ex-4 DMNs

The cytotoxicity of Ex-4 DMNs was evaluated via a MTT cell proliferation assay. HEK293T cells were cultured for 24 h in 3 ml of Dulbecco’s Modified Eagle’s Medium (DMEM), supplemented with 10% foetal bovine serum (FBS) and 1% antibiotics, at a density of 105 cells/well in 6-well plates, at 37 °C, in a humidified atmosphere of 5% CO2. After treating HEK293T cells with 200 μl solutions of blank DMN and Ex-4 DMN dissolved in 200 μl PBS, the cells were incubated for 24 h. Cells were then incubated with 300 μl of MTT reagent (1 mg/ml) for 1 h, then the medium was removed, and the cells were treated with 1 ml of dimethyl sulfoxide (DMSO) per well. Cell viability (%) was calculated at the absorbance of 560 nm using a Victor X5 microplate reader (Perkin Elmer, Waltham, MA, USA). The viability of the PBS-treated control cells was set as 100%.

### Storage stability of Ex-4 DMNs

Each Ex-4 DMN was packed and fully sealed into a plastic bag, with no light exposure, and stored at 4 and 25 °C for 8 weeks. Then, the DMNs were dissolved in 1 ml PBS and activity was measured using UPLC.

### *In vivo* activity of Ex-4 DMNs

Male C57BLKS/J db/db mice (6–7 weeks old) were selected for the Ex-4 bioactivity study. First, the mice were anaesthetised with Avertin (125 mg/kg) and the fur on their backs was removed with a shaver. Before the experiment, the mice were fasted overnight, with free access to water, and randomly divided into the following groups: (a) blank DMN group (negative control); (b) SC injection group (10 μg Ex-4 solution, positive control); (c) Ex-4 DMN patch group (DMNs loaded with 10 μg Ex-4); (d) Ex-4 DMN stored for 8 weeks at 4 °C group (10 μg Ex-4; n = 3 per group). Blood-glucose levels were measured by collecting 20 μl of blood samples from the tail vein at 1, 2, 3, 5, 7, 9, 11, 13, and 15 h post-administration; measurements were conducted using AccuChek Go (Roche Diagnostics, Indianapolis, IN, USA). Plasma was then separated by centrifugation at 13,000 rpm for 10 min. The serum samples were immediately frozen at − 80 °C until analysis. The Ex-4 concentrations in plasma samples were determined using an ELISA kit at the R squared value of 0.97. All animal studies were conducted in accordance with the guide for the care and use of laboratory animals, and in accordance with the procedures approved by the Committee of the Department of Laboratory Animal Medicine (Medical Research Center, College of Medicine, Yonsei University).

### Statistical analysis

All the experimental values are expressed as mean ± S.E. Statistical differences were considered to be significant at a P value < 0.05 (Student’s t-test).

### Data availability

All relevant data are available within the manuscript and supplementary information.

## Electronic supplementary material

Supplementary information
