# Supplementary material for: Exendin-4–encapsulated dissolving microneedle arrays for efficient treatment of type 2 diabetes
Source: Sci Rep. 2018 Jan 18;8:1170. doi: 10.1038/s41598-018-19789-x (PMC5773556; doi:10.1038/s41598-018-19789-x)
Supplement: Supplementary file 1 — Supplementary information [file 41598_2018_19789_MOESM1_ESM.pdf]

Exendin-4–encapsulated dissolving microneedle arrays for efficient treatment of type 2 diabetes

Shayan Fakhraei Lahiji[1,+], Yoojung Jang[1,+], Inyoung Huh[1], Huisuk Yang[1], Mingyu Jang[1,2], Hyungil Jung[1,2,*]

[1] Department of Biotechnology, Building 123, Yonsei University, 50 Yonsei-ro, Seoul 03722, Seodaemun-gu, Korea

[2] Juvic Inc., Building 102, Yonsei Engineering Research Park, 50 Yonsei-ro, Seoul 03722, Seodaemun-gu, Korea

*Corresponding author: hijung@yonsei.ac.kr Phone: 82-2-2123-2884, Fax: 82-2-362-7265

[+]These authors contributed equally to this work.

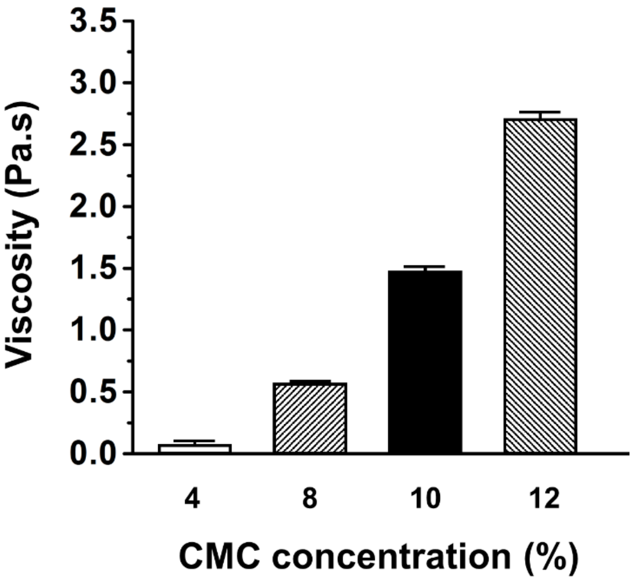

Supplementary Figure 1: Viscosity of CMC at different concentrations. The viscosity of CMC at the concentrations of 4, 8, 10, and 12% (w/v) was measured at the intervals of 200/s. Viscosity is 0.08 Pa.s for 4% CMC, 0.67 Pa.s for 8% CMC, 1.73 Pa.s for 12% CMC, and 3.13 Pa.s for 12% CMC. Increase in the CMC concentration results in increased viscosity.
